# Put Down the ACE: Low Clinical Utility for Angiotensin-Converting Enzyme Levels in Sarcoidosis: A Single-Center Retrospective Cohort Study

**DOI:** 10.3390/jcm13247657

**Published:** 2024-12-16

**Authors:** Amit Druyan, Noam Shuv, Merav Lidar

**Affiliations:** 1 Rheumatology Unit, Sheba Medical Center, Tel Hashomer, Ramat Gan 52621, Israel; 2 Faculty of Medicine, Tel Aviv University School of Medicine, Ramat Aviv, Tel Aviv 69978, Israel; noamshuv@mail.tau.ac.il

**Keywords:** ACE, sarcoidosis, autoimmune disease, clinical epidemiology, diagnostic testing, granulomatous disease

## Abstract

**Background/Objectives**: ACE (angiotensin-converting enzyme) is considered a serological marker of sarcoidosis as elevated levels have been reported in 30–80% of patients. However, elevated ACE levels are also encountered in other medical conditions, and the clinical correlation between ACE levels and disease activity in sarcoidosis is disputable as well. To determine the significance of elevated ACE levels in the diagnosis and follow-up of sarcoidosis patients. **Methods**: All electronic patient records in which an ACE level was recorded in a large tertiary hospital were identified using a computerized algorithm. Medical diagnoses, ACE numerical values, and clinical data were also automatically extracted. Furthermore, all records with a diagnosis of sarcoidosis were manually reviewed for ascertainment of the diagnosis and searched for additional clinical manifestations and treatment responses. **Results**: A total of 1416 records with a documented ACE level were found in the database, and 146 of the records had a diagnosis of sarcoidosis in the medical record. However, the diagnosis was excluded in 27 of these cases after a manual review of the records. Elevated ACE levels were most commonly encountered among patients with sarcoidosis, non-Hodgkin’s lymphoma, cirrhosis, and interstitial lung disease. Elevated ACE levels had a positive predictive value of 12.76% and a negative predictive value of 94.6% for the diagnosis of sarcoidosis in our cohort, with a sensitivity of 63.5% and a specificity of 59.5%. Among patients with sarcoidosis, ACE levels around the time of diagnosis were higher than ACE levels in remission. However, a paired analysis did not find a statistically significant difference in ACE levels between the two timepoints. A positive correlation between lack of cardiac involvement and elevated ACE levels was found on multivariate analysis. **Conclusions**: ACE levels are a non-specific serological marker with low specificity and sensitivity for sarcoidosis and a poor positive predictive value, but with a negative predictive value of 94.6%. Furthermore, elevated ACE levels correlated poorly with disease activity in our cohort.

## 1. Introduction

Sarcoidosis is a multisystem disease of unknown etiology characterized by the infiltration of many organs by non-caseating granulomas. The disease is more prevalent in patients of African American or Scandinavian background, typically manifests between the ages of 30–50, and differs with gender and ethnicity [1,2,3]. Sarcoidosis most commonly involves the lungs, but muscles and skeleton, skin, eyes, liver, spleen, gastrointestinal system, heart, nervous system, kidneys, and other organs may also be involved [1]. Patients with sarcoidosis have a shorter life expectancy than the general population, with most deaths related to sarcoidosis in the Western world attributed to progressive pulmonary fibrosis leading to respiratory failure, pulmonary hypertension, or both [1]. The disease may carry a time-limited or a chronic course.

The estimated incidence of sarcoidosis has a wide range (2.3–11 per 100,000 individuals/year) [4] due to the use of non-uniform diagnostic tools, which typically involve a combination of clinical findings, imaging studies, and biopsy of affected tissue [5], as well as differences in incidence in various ethnic groups, with African American females having the highest incidence of sarcoidosis [6].

Serum angiotensin-converting enzyme (ACE) levels are widely used in the clinic for the diagnosis and monitoring of sarcoidosis, despite accruing evidence of disutility [7]. Potentially, as ACE is produced by epithelial cells in the granuloma, its serum levels may reflect the activity of a granulomatous disease [8]. Previous studies have shown that elevated serum ACE levels have reasonable specificity (83–99%), but low sensitivity (41–100%) [8], and are found in 30–80% of sarcoidosis patients [9]. Furthermore, there are numerous other conditions in which high levels of ACE have been found [9], such as asbestosis, diabetes, Gaucher’s disease, hyperthyroidism, Hodgkin’s lymphoma, lung malignancies, tuberculosis, silicosis, and histoplasmosis. ACE levels are reduced in patients treated with anti-hypertensive drugs belonging to the class of ACE inhibitors.

In the current study, we chose to test again in a relatively large and contemporary cohort of a large tertiary hospital, what is the value of ACE as a biomarker for diagnosis and follow-up of sarcoidosis.

## 2. Materials and Methods

This was a single-center study performed at the Sheba Medical Center—a large tertiary hospital located in central Israel. Data were collected from the computerized patients’ files using MDClone, a data extraction and synthetization platform that provides patient-level data around an index event (http://www.mdclone.com, (accessed on 1 June 2022)). All patient records with a documented serum ACE level between 1 January 2000, and 31 May 2022, were located using the MDClone system to be included in the study. Maximal numeric ACE level results, demographic data, and relevant diagnoses and medications were automatically extracted using the system. For the analysis, maximal documented ACE levels were used. Reference values for sACE did not change throughout the study period. A researcher from the study team then reviewed all patient records with a diagnosis of sarcoidosis to confirm the diagnosis and extract clinical data regarding the manifestations and treatments of sarcoidosis.

The diagnosis of sarcoidosis was established based on accepted criteria, which included either pathological evidence of non-caseating granulomas (with exclusion of other granulomatous diseases and confirmation of systemic involvement) or a specific pathognomonic clinical syndrome [5]. Patients with a documented diagnosis of sarcoidosis who did not meet these criteria were excluded from the study.

Screening for systemic manifestations: As part of the standard care, all patients underwent comprehensive screening for systemic manifestations. Initial screening included a chest X-ray (CXR) for all patients. Those with abnormal CXR findings or respiratory symptoms were further evaluated with a chest CT and pulmonary function tests. Patients presenting with palpable lymphadenopathy, palpable spleen, or systemic symptoms (fever, night sweats, weight loss) underwent either whole-body CT or PET-CT scans. Renal and hepatic involvement were assessed using routine blood and urine analyses.

Cardiac involvement screening included electrocardiograms (ECGs) for all asymptomatic patients. Patients with abnormal ECG findings or clinical indications were referred to cardiac magnetic resonance imaging (MRI). Patients with an implantable cardiac device who could not perform a cardiac MRI were considered to have cardiac involvement of sarcoidosis if no other cause for their electrophysiologic abnormality was found, based on a full cardiologic evaluation. Further evaluations for neurological (central or peripheral), muscular, ocular, or osseous involvement were conducted based on clinical suspicion.

All treatments administered to sarcoidosis patients for the management of the disease were meticulously documented. Prednisone therapy extending beyond 24 months, as well as the necessity for biologic treatments, was extracted from patient records and may indicate a severe disease course. Remission was defined by the treating physician’s determination of the absence of active disease or a recommendation to reduce treatment. A composite end point including the lack of drug-free remission at the end of the follow-up, use of biologics, or chronic prednisone treatment for longer than 24 months, was used as a surrogate for disease severity.

Institutional review board approval was obtained, and patient consent was waived due to the retrospective nature of the study.

Statistical analysis was performed using the JMP 17 pro software (SAS Institute Inc., Cary, NC, USA). Quantitative parameters (age, ACE levels, length of follow-up) were compared using a two-tailed *t*-test, and parametric parameters were compared using a two-tailed Fisher’s exact test. A paired *t*-test was used to compare ACE levels at diagnosis and remission in patients with data at both timepoints. A *p* value of less than 0.05 was considered statistically significant.

Positive and negative predictive values, sensitivity, and specificity were calculated for the diagnosis of sarcoidosis according to elevated ACE levels.

A multivariate Cox proportional hazards regression model was applied to calculate the odds ratios for elevated ACE levels according to different relevant diagnoses and medical treatments. A multivariate Cox proportional hazards regression model was also applied to examine which clinical manifestations correlate with increased ACE levels in sarcoidosis patients. This model was adjusted to age, gender, and the use of ACE inhibitors or angiotensin receptor blockers.

## 3. Results

Using the automatic computerized search, 1416 patient files in which an ACE level test was performed were identified. A diagnosis of sarcoidosis was reported in 146 of the 1416 files. Manual chart review excluded 27 cases after ruling out the diagnosis of sarcoidosis. The remaining 119 patients with a verified diagnosis of sarcoidosis were compared with 1270 patient records without a diagnosis of sarcoidosis in which an ACE level was recorded as well. The sarcoidosis and non-sarcoidosis patients had similar background characteristics as shown in Table 1. Table 2 compares between sarcoidosis patietns with and without elevated ACE levels during their disease course.

Although ACE levels were significantly higher in sarcoidosis patients than in non-sarcoidosis patients (mean 62.3 U/L (SD = 102.8), median 41 [IQR 25.75–67.5] vs. mean 34.9 U/L (SD = 35.7), median 29 [IQR 19–43], *p* < 0.0001), they did not allow for distinguishing between the groups due to overlapping confidence intervals. Indeed, the positive predictive value of increased ACE levels for the diagnosis of sarcoidosis was 12.76%, while the negative predictive value (chance for a negative diagnosis of sarcoidosis in patients with normal ACE levels) was 94.6%. Elevated ACE levels had a sensitivity of 63.5% for the diagnosis of sarcoidosis in our cohort, with a specificity of 59.5%.

**Figure 1:**
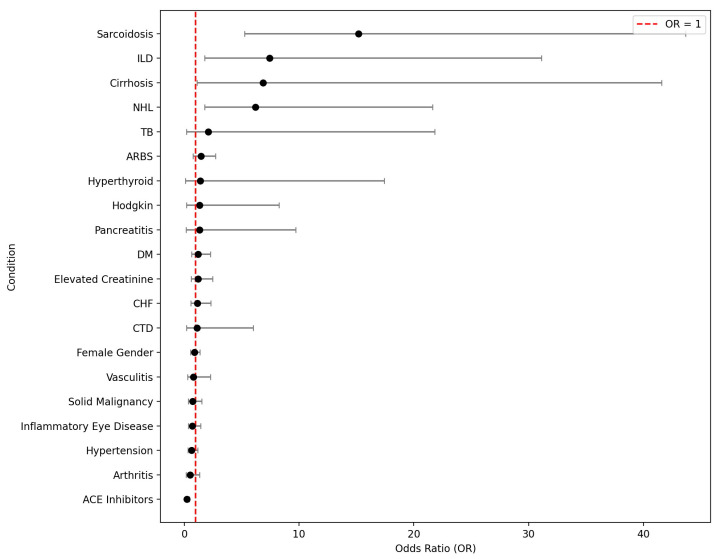
Odds ratios for elevated ACE levels according to diagnosis from a multivariate analysis.

In a univariate analysis, ACE levels were increased in patients with sarcoidosis, lymphoma (Hodgkin’s and non-Hodgkin’s), leukemia, cirrhosis, interstitial lung disease, Gaucher’s disease, hypertension, heart failure, and in those not taking ACE inhibitors. In a multivariate analysis using a Cox proportional hazards regression model, it was found that sarcoidosis, non-Hodgkin’s lymphoma, lack of ACE inhibitor use, cirrhosis, and interstitial lung disease significantly increased the risk of elevated ACE levels. Figure 1 shows the odds ratios for increased ACE levels for the different conditions that were included in the analysis. When excluding 257 patients treated with ACE inhibitors from the analysis, sarcoidosis, non-Hodgkin’s lymphoma, cirrhosis, and interstitial lung disease still significantly increased the risk of elevated ACE levels.

**Figure 2:**
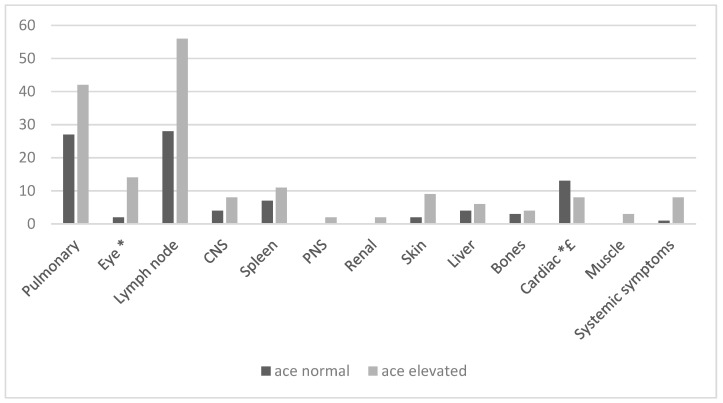
Number of patients with normal and elevated ACE levels according to clinical manifestations. Individual patients may have several manifestations. *—*p* < 0.05 in a univariate analysis. £—*p* < 0.005 in a multivariate analysis.

Figure 2 shows the different symptoms of sarcoidosis in patients according to whether their ACE levels were increased or normal. In a univariate analysis, patients with eye involvement were more likely to have increased ACE levels, and patients with cardiac involvement were more likely to have normal ACE levels. ACE levels did not correlate with any other organ involvement. On multivariate analysis, no organ involvement correlated with elevated ACE levels, whereas normal ACE levels were typically found in patients with cardiac involvement. Methotrexate or biologic agent prescriptions were not correlated with elevated ACE levels. A total of 18/76 patients who ever had an elevated ACE level and 16/43 patients who never had an elevated ACE level were treated with methotrexate (*p* = NS) at some point along their disease course. Moreover, 3/76 patients who ever had an elevated ACE level and 5/43 patients who never had an elevated ACE level were treated with a biologic agent (*p* = NS) at some point along their disease course. Patients with elevated ACE levels at the time of diagnosis were less likely to be treated with corticosteroids for longer than 24 months, compared with patients with normal ACE levels (9/26 vs. 11/15, *p* = 0.003). Moreover, patients who had an elevated ACE level during their disease course were more likely to obtain drug-free remission during their follow-up, with 82.89% of patients with elevated ACE reaching drug-free remission at the end of the follow-up, compared to 60.46% of patients without elevated ACE level during their disease course (*p* < 0.01). When using a composite end point of either use of biologics, chronic corticosteroids use, or lack of drug-free remission at the end of the follow-up as a surrogate for severe disease, patients who had elevated ACE levels at any timepoint during their disease course had less severe disease, compared to patients with normal ACE levels throughout their disease course (48.8% vs. 27.6%, *p* < 0.05). This difference remained significant after excluding patients treated with ACE inhibitors.

A total of 67 patients had an ACE level test at the time of diagnosis. The mean (standard deviation) and median (confidence interval) of the ACE test were 67.26 (110) and 49 (30–72), respectively. Moreover, 67 patients had a record of ACE levels at the time of remission (a median level was taken for each patient), the mean (standard deviation) and median (confidence interval) of the ACE test in remission were 48.05 (59.6) and 33 (23–55), respectively. The mean ACE level at diagnosis was significantly higher, with a *p*-value of 0.0083. Only 18 patients were tested for ACE levels both at diagnosis and in remission. When a paired t-test was performed to test whether there was a difference between the two tests, no statistically significant difference was found (*p* = 0.83), and there was no correlation between ACE levels at diagnosis and ACE levels in remission in these patients.

## 4. Discussion

Serum ACE level testing is regularly used as a supportive measure for the diagnosis and follow-up of sarcoidosis. The data we bring forward, in conjunction with accumulating publications pointing to the low utility of ACE levels in sarcoidosis [7,10], suggest that it is time to set aside this common clinical practice.

A biomarker must exhibit high sensitivity, not be associated with other diseases, be non-invasive, and be available at a low cost. Anti-citrullinated cyclic peptide antibody (anti-CCP) is an exceptional biomarker for rheumatoid arthritis due to its acceptable sensitivity (61.7%) and high specificity (97.1%) owing to its low frequency in other diseases [11]. Our data indicate that while ACE levels have a sensitivity of 63.5% for sarcoidosis, consistent with other studies [7], their specificity is considerably lower at 59.5%, compared to the 93% specificity reported in a meta-analysis [7]. This discrepancy may be due to differences in patient selection between our study and the meta-analysis. Our control group included all patients with documented ACE levels, including those with conditions that elevate ACE levels, such as lymphomas, cirrhosis, and interstitial lung disease. In contrast, some articles in the meta-analysis, particularly those involving prospective trials with healthy controls, excluded these patients. Since lymphomas, liver cirrhosis, and interstitial lung disease are critical to differentiate from sarcoidosis, we believe that patients with these conditions should be included in the control group when calculating the specificity of ACE levels for diagnosing sarcoidosis. Additionally, although average ACE levels in sarcoidosis were higher than in controls, the differences did not allow for clear disease distinction. Specifically, patients with non-Hodgkin’s lymphoma, cirrhosis, interstitial lung disease, and sarcoidosis, all exhibited significantly elevated ACE levels. Sarcoidosis and lymphoma share overlapping clinical and imaging findings, necessitating differentiation due to differing treatments and prognoses. Unfortunately, in this critical clinical situation, ACE levels are unhelpful as they are similarly elevated in both diseases.

Our data show that ACE levels are more commonly elevated in patients with ocular involvement than in those without. As ocular involvement is typically overt, it carries little clinical significance. Unfortunately, ACE levels did not correlate with extra-ocular involvement of sarcoidosis, which is in line with previous studies. Specifically, cardiac involvement, a life-threatening sarcoidosis manifestation, is associated with low rather than elevated ACE levels in our cohort, in concordance with known data in the literature regarding the low sensitivity and specificity of ACE levels in cardiac sarcoidosis [12].

Assessing disease activity in sarcoidosis is challenging, and determining treatment duration is taxing. A distinct change in the level of the biomarker between active disease and remission would be invaluable. Although ACE levels were significantly higher during active disease than in remission, the difference was not significant in the 18 patients with levels available at both timepoints. This small sample size is in line with a meta-analysis showing that ACE levels have moderate performance in predicting sarcoidosis disease activity [7]. An intriguing finding in our cohort is the higher rate of drug-free remission observed at the end of the follow-up period, alongside a reduced rate of chronic prednisone treatment and lower rate (yet statistically not significant) of use of biologics in patients with elevated ACE levels, suggesting that these patients might experience a milder disease course or respond better to treatment. These hypotheses warrant further investigation through larger scale studies.

In this study, we aimed to evaluate the utility of serum ACE levels in diagnosing sarcoidosis and monitoring its clinical course. We found that normal ACE levels had a high negative predictive value (94.6%), while elevated ACE levels demonstrated low positive predictive value, sensitivity, and specificity for diagnosing sarcoidosis. Although ACE levels were higher during active disease compared to remission, individual patients did not show significant changes in ACE levels between active sarcoidosis and remission, a finding that lacks statistical power. Furthermore, elevated ACE levels did not correlate with specific organ involvement and were not predictive of a severe disease course, as indicated by the likelihood of drug-free remission and chronic steroid use. Overall, the findings of our study suggest that ACE testing is not an optimal biomarker for diagnosing, tracking, or assessing the severity of sarcoidosis.

The limitations of our study include its retrospective nature, which prevented us from controlling for all possible factors that might have influenced the results. For example, the reason patients were referred to the ACE level testing is unknown. Although the number of patients with sarcoidosis was relatively small, these were compared to a relatively large number of control patients in which ACE levels were also assessed. This allowed for a sound assessment of specificity as well as positive and negative predictive values of ACE levels in sarcoidosis. Another limitation of our study is that asymptomatic manifestations were not thoroughly evaluated, which may result in the underestimation of these manifestations in our cohort.

Despite these limitations, our study further highlights the issues associated with ACE testing for diagnosing, tracking, and assessing the severity of sarcoidosis. The role of ACE as a universal biomarker for sarcoidosis should be re-evaluated.

## 5. Conclusions

ACE levels are a non-specific serological marker with low specificity and sensitivity for sarcoidosis and a poor positive predictive value. Furthermore, elevated ACE levels correlated poorly with disease activity and disease severity in sarcoidosis in our cohort.

## Figures and Tables

**Table 1:** Baseline characteristics.

	Sarcoidosis Patients (*n* = 119)	Non-Sarcoidosis Patients (*n* = 1270)
Male gender (%)	45.37%	51.18%
Age (mean, STD)	57.01, 14	55.67, 18.8
Elevated sACE (%) *	63%	40.40%
Maximal sACE (mean, STD) *	62.31, 102.83	34.93, 35.74
Diabetes mellitus (*n*)	30	296
HTN	41	410
CHF	14	173
ACE inhibitors (*n*)	22	235
ARBS (*n*)	27	205
Non-Hodgkin lymphoma	1	50
Cirrhosis	3	29
ILD	4	50
Kidney disease	18	214

ACE—angiotensin-converting enzyme; HTN—hypertension; CHF—congestive heart failure; ARBS—angiotensin receptor blockers; ILD—interstitial lung disease. *—*p* < 0.0001.

**Table 2:** Comparison of sarcoidosis patients with and without elevated ACE levels during the disease course.

	Patients with Normal sACE	Patients with Elevated ACE
Number of patients	43	76
Male gender (%)	53.48%	40.78%
Age (mean, STD)	58.51, 12.66	56.16, 14.71
Length of follow-up in months (median, IQR)	55 (38–132)	54 (31–90)
Conditions affecting sACE levels		
ACE inhibitors or ARBs Tx (%)	44%	31%
Non-Hodgkin lymphoma (*n*)	0	1
Cirrhosis (*n*)	0	3
Sarcoidosis manifestations		
Pulmonary involvement (%)	62.80%	55.26%
Eye involvement (%) *	4.65%	18.42%
Lymph node involvement (%)	65.11%	73.68%
CNS involvement (%)	9.30%	10.52%
Spleen involvement (%)	16.28%	14.47%
PNS involvement (%)	0.00%	2.63%
Renal involvement (%)	0.00%	2.63%
Skin involvement (%)	4.65%	11.84%
Liver involvement (%)	9.30%	7.89%
Bone involvement (%)	6.97%	5.26%
Cardiac involvement (%) *	30.23%	10.52%
Muscle involvement (%)	0.00%	3.94%
Systemic symptoms (%)	2.32%	10.52%
Hypercalcemia (%)	2.3%	2.7%
Sarcoidosis treatment		
Methotrexate (%)	37.20%	23.60%
Mercaptopurine (%)	4.65%	5.26%
Infliximab (%)	9.30%	3.94%
IVIG (%)	2.32%	0.00%
Chronic prednisone treatment (%)	71.42%	40%
Treatment-free remission at the end of follow-up *	60.46%	82.89%
Severe disease *	48.8%	27.63%

CNS—central nervous system; PNS—peripheral nervous system; IVIG—intravenous immunoglobulins; Chronic prednisone treatment—defined as more than 24 months of prednisone treatment. Severe disease—defined as either chronic prednisone use, use of infliximab or IVIG, or no treatment-free remission at the end of the follow-up. * *p* < 0.05 in a univariate analysis.

## Data Availability

The data presented in this study are available on request from the corresponding author due to regulatory restrictions.
